# The Incredible Story of India’s Revolutionary Feminist: Dr. Muthulakshmi Reddy (1886–1968)

**DOI:** 10.7759/cureus.62644

**Published:** 2024-06-18

**Authors:** Saranya R, Amrita Ramanathan, Indu Bharkavi SK, Murali Balasubramaniam

**Affiliations:** 1 Department of Oral Pathology, Sathyabama Dental College and Hospital, Chennai, IND

**Keywords:** lawmaker, medical practitioner, educator, social reformer, historical vignette

## Abstract

The term 'trailblazer' is often used casually nowadays, but in the early twentieth century, there was a woman who epitomized it by shattering barriers at every turn. Dr. Muthulakshmi Reddy who holds many firsts to her name was an outstanding Indian woman of her time. She was an eminent medical practitioner who wore multiple hats throughout her life as an educator, a lawmaker, and a social reformer. She sacrificed her lifetime for women's upliftment and children especially the underprivileged. She played a pivotal role in establishing one of India's largest cancer institutes as a ‘mission’ to provide treatment among all sections of people regardless of their socio-economic background. The main purpose of this article is to highlight the indisputable contribution of Dr. Muthulakshmi Reddy in the fields of medicine, education, law, and much more.

## Introduction and background

It is a matter of pride and privilege to be born a woman. This is what the society learns from the distinguished life of Dr. Muthulakshmi. She was a fearless woman who battled against the oppression and subjugation of women. She had many firsts to be remembered such as the first female student admitted to a Men's College, the inaugural woman House Surgeon at the Government Maternity and Ophthalmic Hospital, the pioneering female legislator in British India, the inaugural Chairperson of the State Social Welfare Advisory Board, the first woman Deputy President of the Legislative Council, as well as the first Alderwoman of the Madras Corporation. Throughout her life, Muthulakshmi Reddy remained dedicated to her cause, even in her advanced years. Despite reaching the age of 80, she maintained her vigor and enthusiasm. Her focus shifted from politics to her humanitarian endeavors, steadfastly adhering to her mission and the principles of Gandhism. She was the driving force behind one of India's largest cancer institutes today, the Adyar Cancer Institute. Unfortunately, the remarkable journey of Dr. Muthulakshmi Reddy and her countless milestones against adversity are often overlooked [1,2].

## Review

Early life

**Figure 1:**
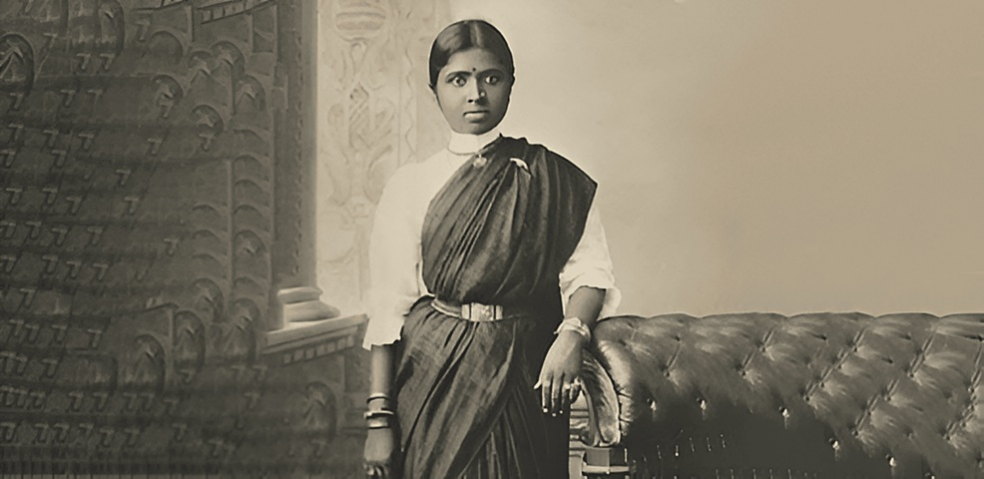
Muthulakshmi Reddy while taking private lessons Credit: Adyar Cancer Institute Archives Permission obtained from Adyar Cancer Institute, Chennai

Originating from modest origins, she was born on July 30, 1886, in Pudukottai, Tamil Nadu, to Narayanaswami Iyer, who served as the principal of Maharaja College at that time, and Chandrammal, formerly a Devadasi. Muthulakshmi Reddy entered the world during a time when the notion of "women should be seen and not heard" was commonly circulated. Yet, Muthulakshmi's determination matched her brilliance; she did not let caste stand in the way of her advancement. Recognizing her keen interest in education, her father resolved to provide her with schooling. From a tender age, her sharp intellect and rapid grasp of concepts propelled her to remarkable success in school. As she matured, she continued her educational journey through home-schooling (Figure 1) [3].

When the time came for her to make pivotal choices, she defied her parents' wishes for an early marriage, opting instead to prioritize her education. Yet, as a woman daring to pursue her academic aspirations, she faced the veiled disapproval of a conservative society. The weight of societal expectations led to the Maharaja College initially rejecting her application despite her stellar academic achievements. It wasn't until Martanda Bhairava Thondaman, the progressive-minded Raja of Pudukkottai, intervened, compelling the college to reluctantly accept her. Thus, shattering the prevalent stereotypes of her time, Muthulakshmi achieved a historic milestone by becoming the inaugural woman to gain admission to Maharajah College, originally reserved for men, in Pudukottai [4].

**Figure 2:**
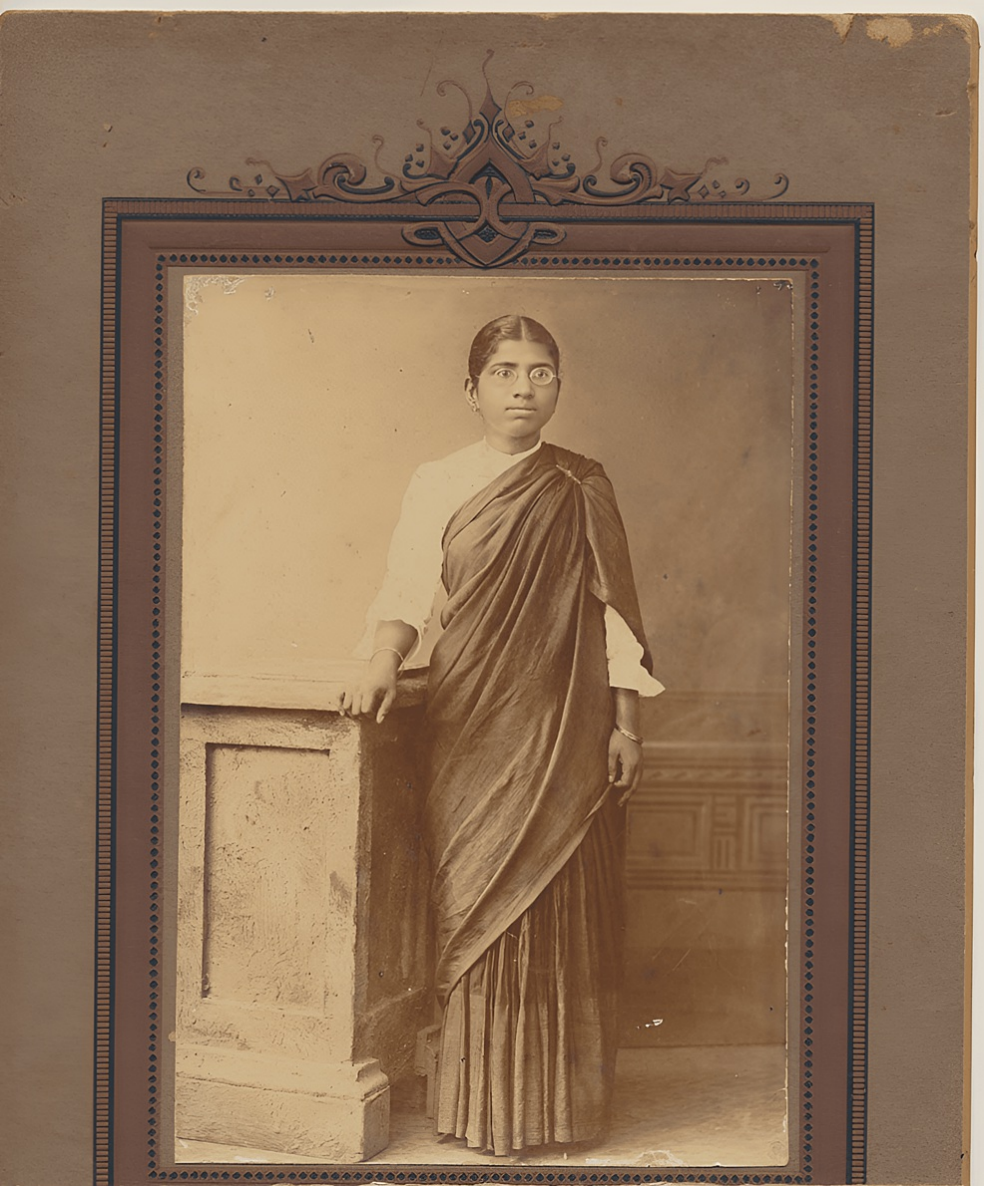
Muthulakshmi Reddy, the medical student Credit: Adyar Cancer Institute Archives Permission obtained from Adyar Cancer Institute, Chennai

However, this was just the outset of her extraordinary journey. Upon completing her undergraduate studies, she pursued admission to Madras Medical College, becoming the sole female candidate in 1907. Subsequently, she made history once again as the first woman House Surgeon at the Government Maternity and Ophthalmic Hospital in Madras (Figure 2).

She pursued her higher education in London. In 1914, she wed Dr. Sundara Reddy, a fellow physician, but she asserted her authority from the outset. She insisted that her husband vow to 'perpetually regard me as an equal and refrain from disregarding my desires.' Dr. Muthulakshmi, as a physician, initially prioritized challenging the practice of 'wet nursing', which involved affluent women hiring Dalit women to breastfeed their babies, as childcare and breastfeeding were considered unsuitable tasks for privileged women. She raised awareness among her patients about the advantages of breastfeeding infants with their mother's milk [2,5].

As a freedom fighter and legislator

The passion of the Indian freedom movement ignited her spirit, and Mahatma Gandhi’s compelling leadership deeply influenced her. Guided by his example, she actively participated in the struggle for independence, collaborated with fellow leaders, and made significant contributions to the cause. Dr. Muthulakshmi collaborated with theosophist and advocate for women's rights, Annie Besant. Together with Besant and a small group of like-minded individuals, she established the Women's Indian Association (WIA) in 1917. Being one of the rare female leaders in South India, she passionately campaigned for India's freedom from colonial rule. In 1932, Dr. Muthulakshmi, alongside Gandhiji, represented India at the Third Round Table Conference in London, gaining considerable visibility. Later, in 1934, she took part in the First International Conference in Chicago [6].

After the passing of Annie Besant, she took on the presidency of the WIA, thereby becoming the first female legislator in India. This milestone marked the commencement of Dr. Muthulakshmi's lifelong endeavor to rectify gender disparities by combating social injustices and advocating for equality. Her book, My Experience as a Legislator, documents all of her contributions to the Legislature. Her most commendable achievement as a legislator was the passing of the resolution abolishing the Devadasi system. She also passed a resolution to establish a special hospital for women and children and the Kasturba Hospital at Triplicane is a monument to her efforts. Further, she recommended systematic medical inspection of students in all schools and colleges. She passed the bill for the suppression of brothels and immoral trafficking in women and children and a rescue home for the same was opened through her efforts. She advocated for raising the legal marriage age and played a pivotal role in establishing women's toilets. Additionally, she spearheaded efforts to enhance medical services for residents of slum areas. Serving as a Member of the Legislative Council, she implemented a program offering free education for girls up to the eighth grade. Furthermore, she worked to lower fees for secondary education for underprivileged girls and secured grants for institutions aimed at training adult women, among other initiatives [7,8].

Birth of the Avvai Home, Orphanage and School

In the twilight of a June evening in 1930, three young Devadasi girls sought refuge at her doorstep. Finding no sanctuary elsewhere, she endeavored to secure their admission into hostels and schools. The hostels were all segregated by caste and would not accept them, and the schools also refused to admit them. This prompted her to take matters into her own hands by providing them with both shelter and education. This led to the creation of 'Avvai Illam' (Avvai's Home) for poor and destitute girls in 1931. One of the girls later became a teacher, another one a doctor, and the third a staff nurse. Initially established to safeguard and educate liberated girls from the Devadasi community, Avvai Home evolved into a haven for all women and children regardless of social or caste distinctions. Presently, Avvai Home boasts an educational complex encompassing a school and a teacher training institution, which serve as a platform for empowering and fostering economic independence among underprivileged girls and women [2,9,10].

The Cancer Institute (WIA)

In the bustling expanse of Chennai, the Adyar Cancer Institute stands as a beacon of hope, its towering structure a testament to its profound impact. Since its establishment, this multistory edifice has been a sanctuary for countless individuals whose lives were besieged by the ravages of cancer. Dr. Reddy's narrative would lack essential depth without delving into the genesis of her bravery in founding the Cancer Institute. As a fresh medical graduate, Dr. Reddy bore witness to the anguish, suffering, and eventual demise of her sister due to a misdiagnosed instance of rectal cancer. During the era of British colonial rule in India, resources for cancer treatment were virtually non-existent. Her dream to create a cancer facility where care would be extended regardless of social or economic status met many obstacles due to public ignorance regarding cancer and governmental indifference. When she petitioned the Government of Tamil Nadu for land, she was bluntly questioned, "Why a Cancer Hospital? People only die of cancer" [10].

Yet, Dr. Muthulakshmi Reddy did not give up on her dream, and with the help of WIA, the Adyar Cancer Institute came into existence in 1954 in the form of a small hut. Today, the Adyar Cancer Institute boasts over 650 hospital beds of national and international stature, the pillars behind it being Dr. S Krishnamurthy (son of Dr. Muthulakshmi Reddy) and Dr. V Shanta. Shanta referred to Muthulakshmi as "mother" and considered Krishnamurthy her mentor. As his dedicated colleague, she worked alongside him to guide the institute in embodying Muthulakshmi's values and ethos, all while striving for excellence. The Adyar Cancer Institute now comprises a comprehensive research division, a preventive Oncology department, and the esteemed Dr. Muthulakshmi College of Oncologic Sciences. Established as a voluntary, charitable, non-profit organization funded by public donations, it has remained faithful to its principle of "Service to all." Its services are provided either free of charge or at a subsidized rate. Presently, it stands as a globally recognized institution, which has been rated by the WHO as the top-ranking cancer center in the country [10,11].

Recognitions and award

Dr. Reddy’s efforts to empower women and children, particularly those in underserved communities, were acknowledged in 1947 when the first national flag was hoisted at Red Fort, bearing her name. The Tamil Nadu government has declared that Hospital Day celebrations will take place annually on July 30, commemorating the birth anniversary of one of the nation's successful female doctors from the early twentieth century. The Government of Tamil Nadu also introduced Dr. Muthulakshmi Reddy Maternity Benefit Scheme for the well-being of pregnant women and newborns. In acknowledgment of her significant contributions, she was bestowed with the Padma Bhushan, India's third-highest civilian honor, in 1956. Google Doodle celebrated the 133^rd^ birth anniversary of Dr. Muthulakshmi, one of India's first female doctors and the country's first woman legislator [4,6,10].

## Conclusions

Muthulakshmi Reddy persisted in her fight for her cause until the very end of her life, never allowing obstacles to deter her. Achieving numerous firsts as a woman in India, she breathed her last at the age of 81 on July 22, 1968. Although the saint is no longer with us, her legacy endures in every strong woman who advocates for education and equality. The living monuments to her memory are the Avvai Home and the Adyar Cancer Institute. My article is a tribute to honor her meaningful life that has made an impact on millions of people making her immortal.

## References

[1] Muthulakshmi Reddy S (1930). My Experience as a Legislator. https://amritmahotsav.nic.in/unsung-heroes-detail.htm?2703.

[2] Dr Muthulakshmi Reddy (2024). Dr Muthulakshmi Reddy: a pioneering feminist. A Pioneering Feminist, Aditi Shah, 7th December.

[3] (2024). Landmarks in Indian history: Dr Muthulakshmi Reddi. https://artsandculture.google.com/story/landmarks-in-indian-history-dr-muthulakshmi-reddi-zubaan/FwXR0CInsH82Ig?hl=en.

[4] (2024). The inspiring story of Dr Muthulakshmi Reddy, who broke barriers in education, medicine and law. https://www.dailyrounds.org/blog/not-just-a-doctor-the-inspiring-story-of-dr-muthulakshmi-reddy/.

[5] (2024). Giants in history: Muthulakshmi Reddy. https://www.asiaresearchnews.com/content/muthulakshmi-reddy.

[6] Regi S (2019). Contributions of Dr. S. Muthulakshmi Reddy. Int J Res Anal Rev.

[7] (2024). Dr Muthulakshmi Reddy: the unsung feminist of India. https://www.indiatoday.in/education-today/gk-current-affairs/story/dr-muthulakshmi-reddy-the-unsung-feminist-of-india-1575138-2019-07-30.

[8] Santhi S, Saravanakumar AR (2020). Contribution of Dr. Muthulakshmi Reddy to women empowerment -a historical study. International Journal of Scientific and Technology Research.

[9] Devika VR (2022). Muthulakshmi Reddy: A Trailblazer in Surgery and Women's Rights. https://niyogibooksindia.com/books/muthulakshmi-reddy/.

[10] Shanta V (2012). Muthulakshmi Reddy: A Legend Unto Herself.

[11] Harish K, Gopinath KS (2021). V Shanta - tribute to the legend and a saint. Indian J Surg Oncol.

